# Ellagic acid protects mice against sleep deprivation-induced memory impairment and anxiety by inhibiting TLR4 and activating Nrf2

**DOI:** 10.18632/aging.103270

**Published:** 2020-05-20

**Authors:** Wenjun Wang, Liudi Yang, Tianlong Liu, Jingwen Wang, Aidong Wen, Yi Ding

**Affiliations:** 1 Department of Pharmacy, Xijing Hospital, Fourth Military Medical University, Xi’an 710032, China; 2 College of Pharmacy, Shaanxi University of Chinese Medicine, Xianyang 712046, China; 3 Department of Rehabilitation Medicine, Xi'an International University, Xi’an 710077, China

**Keywords:** sleep deprivation (SD), ellagic acid (EA), memory impairment, Nrf2, TLR4

## Abstract

Sleep disorder has become a prevalent issue in current society and is connected with the deterioration of neurobehaviors such as mood, cognition and memory. Ellagic acid (EA) is a phenolic phytoconstituent extracted from grains and fruits that has potent neuroprotective properties. This research aimed to study the alleviative effect and mechanism of EA on memory impairment and anxiety caused by sleep deprivation (SD). EA ameliorated behavioral abnormalities in SD mice, associated with increased dendritic spine density, and reduced shrinkage and loss of hippocampal neurons. EA reduced the inflammatory response and oxidative stress injury caused by SD, which may be related to activation of the Nrf2/HO-1 pathway and mitigation of the TLR4-induced inflammatory response. In addition, EA significantly reduced the mortality and ROS levels in glutamate (Glu)-induced hippocampal neuron injury, and these effects of EA were enhanced in TLR4 siRNA-transfected neurons. However, knockdown of Nrf2 dramatically restrained the protective impact of EA on Glu-induced toxicity. Taken together, EA alleviated memory impairment and anxiety in sleep-deprived mice potentially by inhibiting TLR4 and activating Nrf2. Our findings suggested that EA may be a promising nutraceutical ingredient to prevent cognitive impairment and anxiety caused by sleep loss.

## INTRODUCTION

Sleep is a fundamental conserved physiological process of the human body, and the quality and quantity of sleep can affect individual health status and quality of life [1]. Although people are advised to sleep seven to nine hours every day, there is a high prevalence of insufficient sleep in modern societies [2]. In particular, sleep disorders are common with aging [3]. It has been well documented that inadequate sleep is detrimental to human health [4, 5]. The traditional way to study sleeplessness and its consequences is sleep deprivation through sensory stimulation [6]. Sleep deprivation has long been known to impair neurobehavior. This cognitive function impairment is related to increased oxidative stress and inflammation in the brain. In particular, the hippocampal region of the brain appears to be more susceptible to SD than other areas. In addition, the median concentrations of glutamate (Glu) were higher during SD compared to baseline in rats [7]. Glu generates oxidative stress by various mechanisms, which leads to increased reactive oxygen species (ROS) production. The abnormal production of ROS causes oxidation of biological macromolecules and the expression of inflammatory mediators and genes, ultimately leading to an increase in the risk of neurodegenerative diseases.

Toll-like receptor (TLR) activation plays an important role in regulating the innate immune response against exogenous pathogens, endogenous risk factors and immune disorders [8]. Twelve members of the TLR family have been identified in mammals, of which TLR4 is expressed on the cell surface. Nuclear factor erythroid 2-related factor 2 (Nrf2) is known as a critical regulator of endogenous inducible defense systems in the brain to defend against oxidative stress. Under normal conditions, Nrf2 localizes to the cytoplasm and binds to Kelch-like ECH-associated protein 1 (Keap-1), which mediates its proteasomal degradation, whereas Nrf2 activation induces its translocation to the nucleus to regulate its downstream enzymes, such as heme oxygenase 1 (HO-1) [9]. Simultaneously, Nrf2 signaling also plays an important role in the modulation of inflammatory responses. Numerous studies have demonstrated that TLR4-mediated innate immune responses and the Nrf2-modulated antioxidant system may coordinate in various ways to regulate inflammation [10, 11].

Polyphenols are products of plant metabolism, and reports from epidemiological investigations have suggested that ingestion of phenolic foods may reduce age-associated neurodegeneration, such as Alzheimer’s disease [12, 13]. Ellagic acid (2,3,7,8-tetrahydroxy-benzopyranol (5,4,3-cde) benzopyran-5,10-dione), a natural polyphenolic bioactive and lactone compound, is produced in plants, nuts and fruits [14, 15]. Its molecular formula is C_14_H_6_O_8_ (Figure 10A) [16]. EA has a variety of pharmacological properties including anti-inflammatory, antioxidant and neuroprotective activities [17]. EA is also classified as nutraceutical because of its significant health-promoting bioactivities [18]. Several studies have shown that EA influences a series of signal mechanisms to decrease the development of certain neurodegenerative anomalies [19]. Nevertheless, so far, the influence of EA on behavioral functions in experimental models of SD has not yet been elucidated. Moreover, there is no report on the effects of EA on memory deficits and anxiety caused by SD.

Considering all of the abovementioned points, this study was designed to examine the preventive effects of EA on memory deficits and anxiety induced by SD and to determine whether these neuroprotective effects were modulated by the Nrf2 and TLR4 pathways in the brain.

## RESULTS

### EA improved learning and memory in SD mice

**Figure 1:**
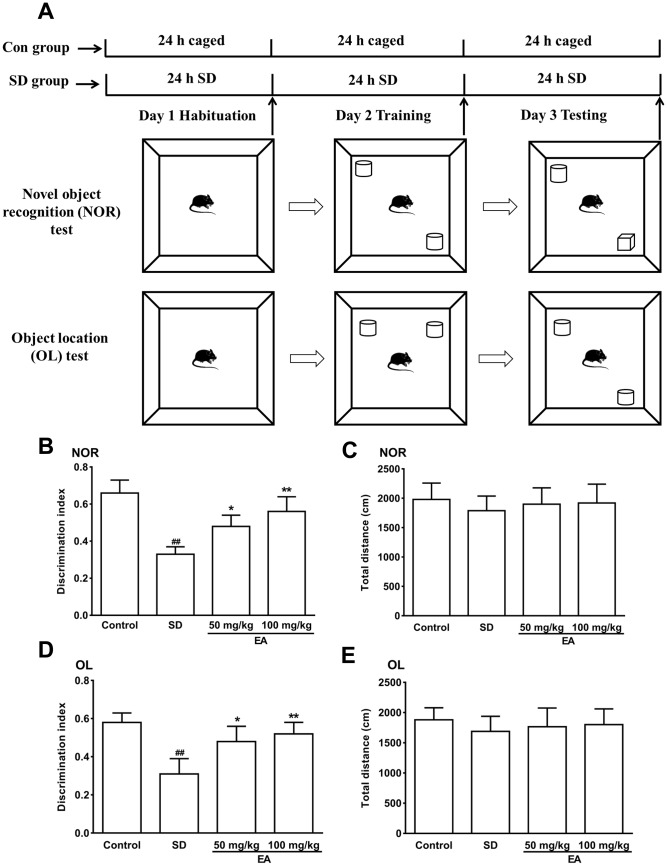
The novel object recognition (NOR) and object location test (OL) test performances were shown in **A**–**C**. (**A**) Schematic of the NOR and OL tests. (**B**) Discrimination index toward a novel object and (**C**) total distance travelled (during 10 min test) were summarized. (**D**) The discrimination index toward a novel location and (**E**) total distance traveled (during the 10-minute test) were summarized. Data values were expressed as the mean ± SEM (n=12), ^##^*P* < 0.01 vs. control group; ^*^P < 0.05 and ^**^P < 0.01 vs. SD group.

Mice were deprived of sleep for 72 hours and subjected to behavioral testing 24 hours later (Figure 1A). SD mice showed disinterest in exploring the novel object. Compared to control group mice, SD mice were also observed to devote less time to exploration overall, and the number of times they explored new objects was significantly reduced (*P* < 0.01), indicating that SD impaired the recognition memory of mice. However, the EA group mice devoted even more time to exploration of the object, and the frequency of exploring the new object was considerably higher than that in the SD group mice (*P* < 0.05, *P* < 0.01, Figure 1B). Similarly, SD mice showed impairments in the novel location test, which was utilized to determine the capacity of the mice to remember the locations of objects. In addition, the EA groups took more opportunities to acquire the object, with more episodes of exploring new objects compared to the SD group (*P* < 0.05, *P* < 0.01, Figure 1D). To exclude the interference of locomotor activity, the total distance traveled was analyzed. No significant difference in the total distance was found between the control and SD groups or between the SD and EA groups (*P* > 0.05, Figure 1C, 1E).

**Figure 2:**
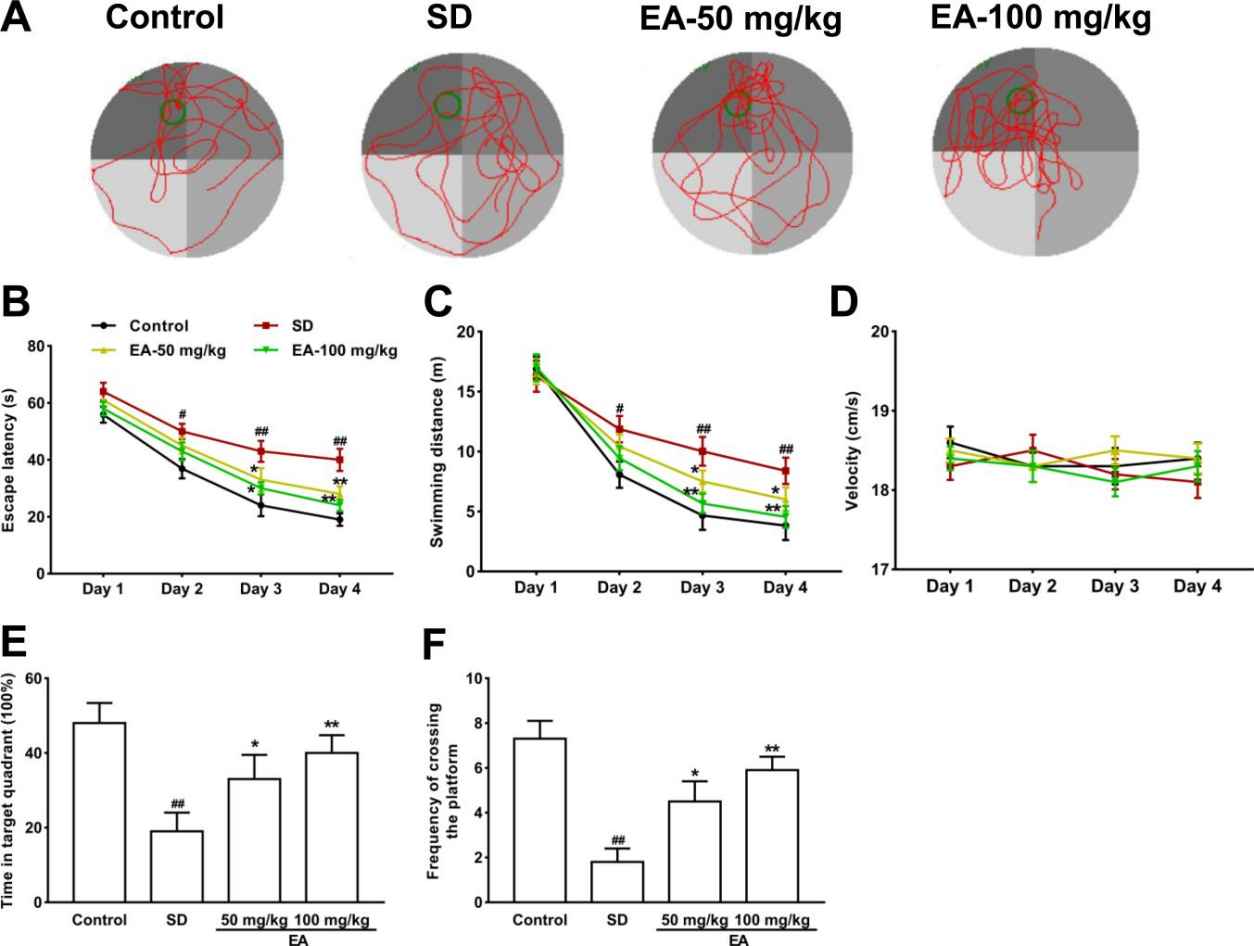
**Effect of CL on spatial reference memory in the MWM test in mice.** (**A**) Representative swimming tracks in the MWM during the probe trial. (**B**) Mean daily escape latencies (time from the start to the hidden platform). (**C**) Distance travelled during the learning phase of the water maze task. (**D**) The swimming velocity of the mice. (**E**) The percentage of time spent in the target quadrant during the probe trial. (**F**) Frequency of crossing the target quadrant during the probe trials. All values were expressed as the mean ± SEM (n=12), ^#^*P* < 0.05 and ^##^*P* < 0.01 vs. Control group; ^*^P < 0.05 and ^**^P < 0.01 vs. SD group.

In the MWM test (Figure 2A), the escape latency to the platform and total swimming distance were noticeably decreased in all groups except for the SD group from the second day of the training phase (Figure 2B, 2C). Moreover, the swimming velocity of the mice was similar among groups, indicating the intact locomotor activity of mice (*P* > 0.05, Figure 2D). The above results indicated that the sleep-deprived mice could not remember the location of the target platform. Subsequent comparison showed significant differences in the time in the target quadrant and the frequency of crossing the platform between groups during the probe trials (*P* < 0.05, Figure 2E, 2F). The shorter time in the target quadrant in the SD group was obviously reversed by EA, and the platform crossing times were also increased with EA (*P* < 0.05, *P* < 0.01, Figure 2E, 2F). These results indicated that EA could alleviate SD-induced learning and memory deficits.

### EA alleviated anxiety-like behaviors in SD mice

**Figure 3:**
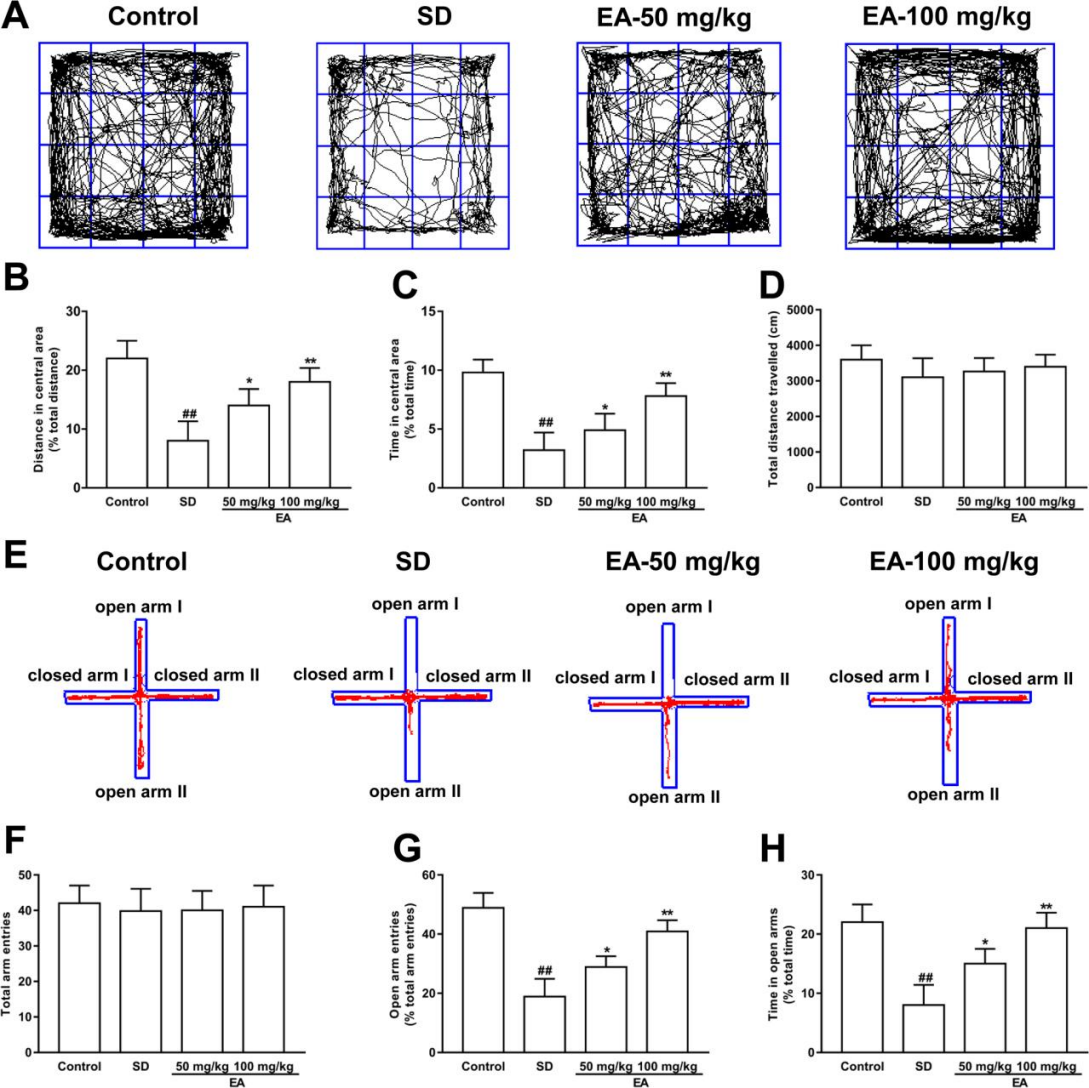
**Effect of CL on sleep deprivation induced anxiety-like behaviors.** (**A**) Sample traces of locomotor activity in the open field test. (**B**) The total distance traveled and (**C**) time spent in the center area. (**D**) The total distance traveled (during the 15-minute test) was summarized. (**E**) Sample traces of locomotor activity in the elevated plus maze test. (**F**) The total arm entrances. (**G**) The entrance into the open arms and (**H**) time spent in the open arms. Data values were expressed as the mean ± SEM (n=12), ^##^*P* < 0.01 vs. control group; ^*^P < 0.05 and ^**^P < 0.01 vs. SD group.

Open field and EPM tests were utilized to check anxiety-like behaviors. Both the distance traveled and the time spent in the center location were reduced in sleep-deprived mice in the open field test (*P* < 0.01, Figure 3A). Nevertheless, the shorter time in the central region in the SD group was obviously reversed by EA, and the total distance traveled was also increased with EA (*P* < 0.05, *P* < 0.01, Figure 3B, 3C). No difference in total distance traveled was found between the SD and EA groups (*P* > 0.05, Figure 3D). Similarly, no difference in overall entrance to the open and closed arms was found among all groups in the EPM test (*P* > 0.05, Figure 3E, 3F). Moreover, the number of entrances into the open arms and the time spent in the open arms were significantly decreased in the SD mice (*P* < 0.01). However, these results were improved significantly when SD mice were given EA (*P* < 0.05, *P* < 0.01, Figure 3G, 3H), which indicated that EA could alleviate SD-induced anxiety-like behaviors.

### EA improved neuron survival in sleep-deprived mice

**Figure 4:**
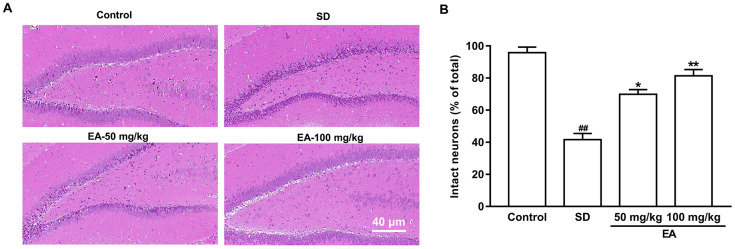
**EA improved neuronal survival after SD.** (**A**) The hippocampus was stained by hematoxylin and eosin. (**B**) The percentage of intact neurons relative to the total neurons for each group (six different fields were counted per slice). Scale bar=40 μm. Data values were expressed as the mean ± SEM (n=3), ^#^*P* < 0.05 and ^##^*P* < 0.01 vs. control group; ^*^P < 0.05 and ^**^P < 0.01 vs. SD group.

Neurons of a certain number and normal function are important for animals. The normal neurons in the hippocampus from the control group were packed tightly and orderly with clear nuclei. In contrast, the SD group exhibited obvious pathological abnormalities with loosely arranged neurons, pyknotic nuclei and loss, or dark color staining in the hippocampus, suggesting that the neurons began to degenerate. However, these histopathological alterations were dramatically attenuated after EA administration (Figure 4A). The numbers of normal neurons were significantly decreased to 41.6 ± 3.88% compared with 95.79 ± 3.53% in the control group (*p* < 0.01). Normal cells were significantly increased to 69.85 ± 2.98% and 81.36 ± 3.92% in the two EA-treated group, respectively (*P* < 0.05, *P* < 0.01, Figure 4B).

### EA restored dendritic spine density in the hippocampus

**Figure 5:**
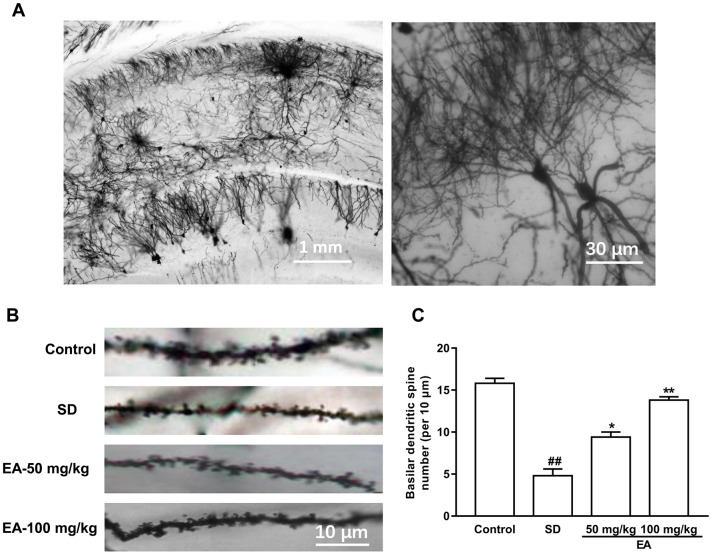
**EA treatment reversed the spine density in the hippocampus area.** (**A**) Golgi-Cox staining of CA1 pyramidal neurons for spine counting. (**B**) Representative images of basilar dendrites and (**C**) summary of spine counts from basilar dendrites. Data values were expressed as the mean ± SEM (n=3), ^##^*P* < 0.01 vs. control group; ^*^P < 0.05 and ^**^P < 0.01 vs. SD group.

Dendritic spines are the basic structural units that underlie the learning and memory formation. Given the role of EA in histological changes and behaviors, we predicted that EA would affect spine density (Figure 5A). Consistent with this hypothesis, SD significantly decreased spine density in the CA1 area (*P* < 0.01), and administration of EA significantly increased spine density in SD mice (*P* < 0.05, *P* < 0.01, Figure 5B, 5C).

### Effects of EA on proinflammatory cytokine levels and oxidative stress parameters

To investigate the effects of EA on oxidative stress and the inflammatory response caused by SD, we tested the expression of related oxidative stress parameters and inflammatory factors. As shown in Table 1, the activities of GPx and SOD were markedly decreased in the hippocampus of the SD group compared to those in the hippocampus of the control group (*P* < 0.01). The administration of EA resulted in a significant increase in SOD and GPx activities (*P* < 0.05, *P* < 0.01). The MDA content was significantly increased in the SD group compared to that in the control group (*P* < 0.01), while EA caused a significant reduction in the MDA content (*p* < 0.05). The levels of 3 proinflammatory cytokines released were significantly increased in the hippocampus of the SD group (*P* < 0.05, *P* < 0.01), while administration of EA significantly decreased proinflammatory cytokine levels (*P* < 0.05, *P* < 0.01, Table 2). These results indicated that EA have effectively controls proinflammatory cytokine levels and antioxidant enzyme activities caused by SD in the hippocampus. Furthermore, the high-dose EA had stronger activities than the low-dose EA.

**Table 1:** Levels of SOD, GPx and MDA in the hippocampus after SD in each group.

**Groups**	**SOD U/mg**	**GPx U/mg**	**MDA μmol/mg**
Control	116.7 ± 11.7	91.4 ± 6.1	1.2 ± 0.2
SD	58.5 ± 4.9^##^	36.8 ± 4.2^##^	2.8 ± 0.4^##^
EA (50 mg/kg)	84.8 ± 6.4^*^	62.2 ± 5.8^*^	2.0 ± 0.2
EA (100 mg/kg)	106.9 ± 9.8^**^	81.4 ± 9.4^**^	1.8 ± 0.1^*^

Data values were expressed as the mean ± SEM (n=3), ^##^*P* < 0.01 vs. control group; ^*^P < 0.05 and ^**^P < 0.01 vs. SD group.

**Table 2:** Levels of IL-1β, IL-6 and TNF-α in the hippocampus after SD in each group.

**Groups**	**IL-1β pg/mg**	**IL-6 pg/mg**	**TNF-α pg/mg**
Control	26.7 ± 1.2	59.2 ± 3.3	28.8 ± 2.2
SD	49.4 ± 2.8^#^	99.7 ± 8.2^##^	56.5 ± 3.1^##^
EA (50 mg/kg)	35.3 ± 2.0^*^	76.7 ± 5.6^*^	42.2± 1.7^*^
EA (100 mg/kg)	33.9 ± 0.9^*^	63.1 ± 3.2^**^	32.5 ± 1.1^**^

Data values were expressed as the mean ± SEM (n=3), ^#^*p* < 0.05 and ^##^*P* < 0.01 vs. control group; ^*^P < 0.05 and ^**^P < 0.01 vs. SD group.

### EA modulated the Nrf2/HO-1 and TLR4-induced inflammatory responses

**Figure 6:**
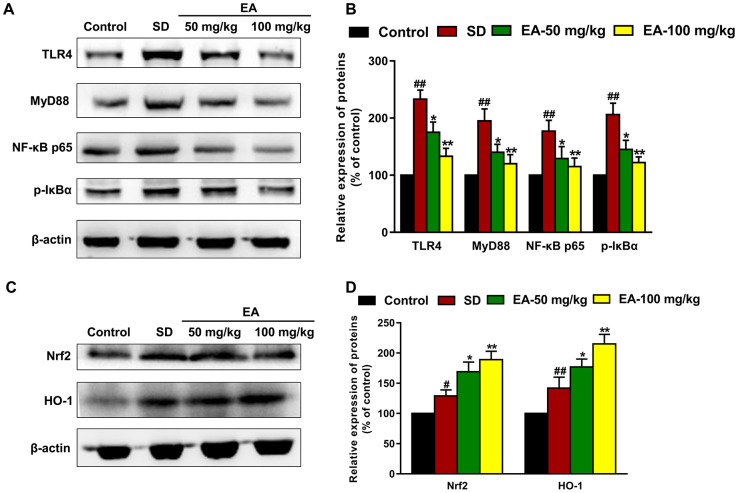
**EA modulated the Nrf2 and TLR4 signaling pathways.** (**A**) and (**C**) The levels of Nrf2, HO-1, TLR4, MyD88, p-IκBα and NF-κB p65 in the hippocampus were detected by Western blot. (**B**) and (**D**) Band intensities were quantified as percentages of values from the control group. Data values were expressed as the mean ± SEM (n=3), ^#^*P* < 0.05 and ^##^*P* < 0.01 vs. control group; ^*^P < 0.05 and ^**^P < 0.01 vs. SD group.

Nrf2-ARE is an endogenous inducible defense system that defends against oxidative stress, and TLR-induced signaling pathways are the main pathway leading to inflammatory responses in the brain. Thus, we investigated the expression of Nrf2, HO-1, TLR4, MyD88, p-IκBα and NF-κB p65 in different groups using western blotting (Figure 6A, 6C). SD mice showed a significant enhancement in Nrf2 and HO-1 immunoactivity (*P* < 0.01) and an increase in the expression of TLR4, MyD88, p-IκBα and NF-κB p65 compared to the control group (*P* < 0.01). However, EA effectively activated the Nrf2/HO-1 pathway and downregulated the TLR4-induced inflammatory response (*P* < 0.05, *P* < 0.01, Figure 6B, 6D).

### Protective effects of EA on glutamate-induced toxicity

**Figure 7:**
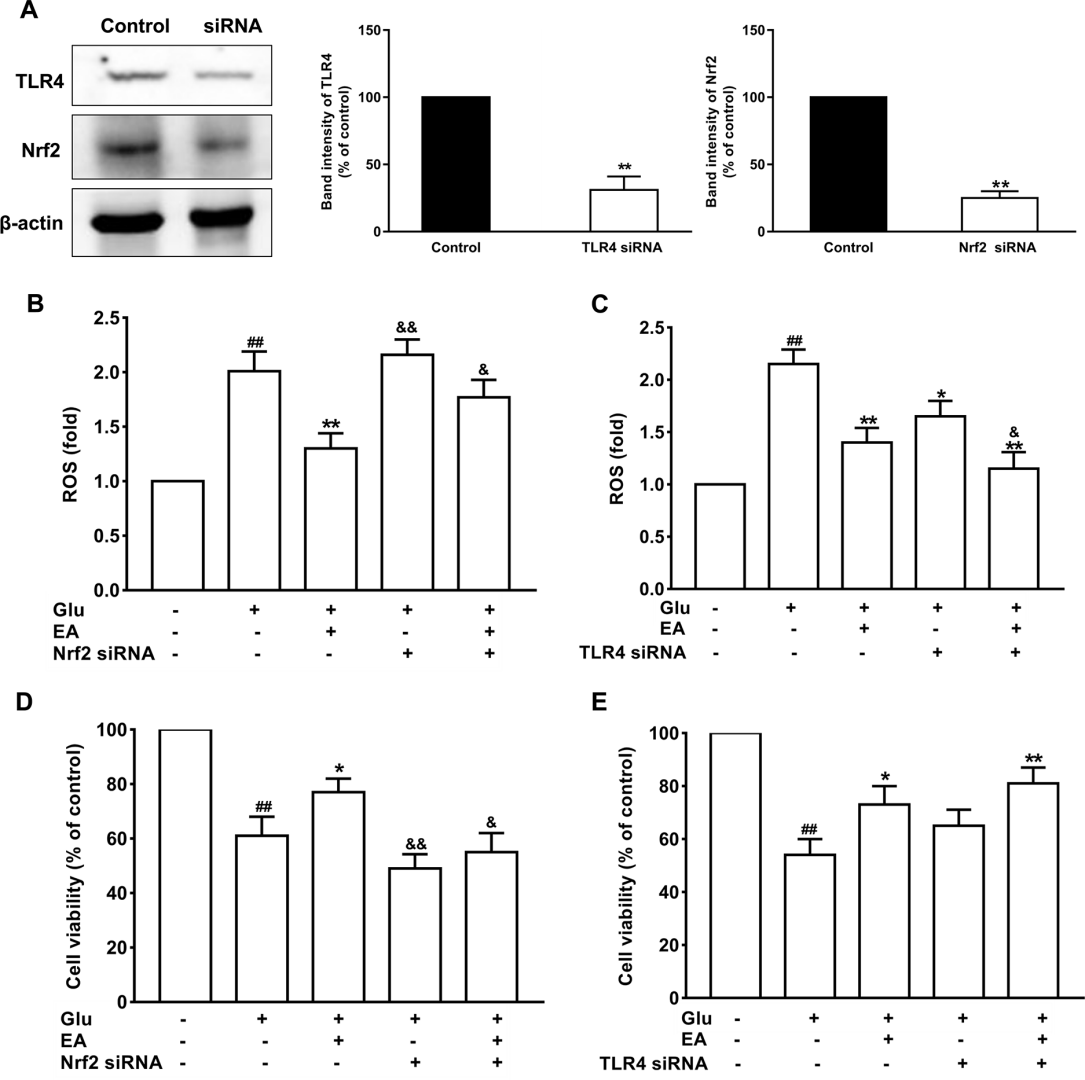
**The protective effects of EA on glutamate-induced toxicity in neuronal cells.** (**A**) The expression levels of Nrf2 and TLR4 significantly decreased in the siRNA treatment group. (**B**) and (**C**) Effect of EA on ROS levels in Nrf2 or TLR4 siRNA-transfected and Glu-treated neuronal cells. (**D**) and (**E**) Effect of EA on cell viability in Nrf2 or TLR4 siRNA-transfected and Glu-treated neuronal cells. Data values were expressed as the mean ± SEM (n=3), ^##^*P* < 0.01 vs. control group; ^*^P < 0.05 and ^**^P < 0.01 vs. Glu group; ^&^*P* < 0.05 and ^&&^*P* < 0.01 vs. EA-treated Glu group.

Primary hippocampal neuronal cells were transfected with Nrf2 or TLR4 siRNA to further verify the protective effect of EA and the involvement of the Nrf2 and TLR4 signaling pathways in Glu-induced toxicity. As shown in Figure 7A, the results showed that treatment with Glu led to obvious expression of Nrf2 and TLR4 in hippocampal neuronal cells, while the expression levels of Nrf2 and TLR4 significantly decreased in the siRNA treatment group (*P* < 0.01), indicating successful transfection. Glu significantly increased ROS production and the rate of neuronal apoptosis (*P* < 0.01), and this effect was partially enhanced by Nrf2 knockout and suppressed by TLR4 knockout (*P* < 0.05, Figure 7B–7E). Moreover, EA significantly reduced neuronal mortality and ROS levels (*P* < 0.01), and these effects of EA were enhanced in TLR4 siRNA-transfected neurons (*P* < 0.01, Figure 7C, 7E). However, knockdown of Nrf2 dramatically restrained the protective impact of EA on Glu-induced toxicity, as illustrated by the absence of recovery of ROS and cell viability in the siRNA-treated Glu group (*P* < 0.01, *P* < 0.05, Figure 7B, 7D).

## DISCUSSION

A previous report showed that the neuroprotective effects of EA has improved cognitive behavior in rats with traumatic brain injury [20]. However, whether EA can ameliorate cognitive impairment and emotional disorders has not been clearly demonstrated. The present research supported our hypothesis that EA treatment ameliorates behavioral abnormalities induced by SD. We also found that the potential molecular mechanism for the effects of EA versus SD was related to the modulation of TLR4 and Nrf2. Furthermore, EA treatment reversed the dendritic spine loss caused by sleep deprivation. Consistent with the behavioral data in our study and others, EA had a normalizing effect on the levels of proinflammatory cytokines and oxidative stress parameters in the hippocampus of SD mice. Thus, this study suggests that EA may be a prospective candidate for the prevention of SD-induced behavioral abnormalities.

Sleep is a restorative process that facilitates learning and memory consolidation [21]. SD is understood to impair both emotional and contextual memories by modifying the neuronal network at physiological, molecular, and synaptic levels [22]. In our present study, EA significantly improved spatial memory impairment in mice after 72 hours of sleep deprivation. Furthermore, EA obviously reduced the shrinkage and loss of hippocampal neurons in SD mice. The dendritic spine density was dramatically increased after EA administration. We found that the reduction in the inflammatory response and oxidative stress injury may be related to EA's protection of mice from memory impairment and anxiety caused by SD. However, how EA modulates the inflammatory response and oxidative stress remains unknown.

EA has long been reported to have a strong neuroprotective effect [23], but no studies have yet reported its effects on the memory impairment and anxiety induced by SD via the Nrf2 and TLR4 pathways. Nrf2 regulates the antioxidant system and can be activated in response to oxidative stress. Our laboratory has previously demonstrated that dietary EA can act as an antioxidant via Nrf2 activation [24]. Nrf2 overexpression shows neuroprotective effects [25]. ROS overproduction is recognized as having the ability to activate Nrf2 by degrading its associated protein, Keap1. Then, Nrf2 migrates to the nucleus and promotes the expression of proteins such as HO-1 [26]. TLR signaling can be affected by the cellular redox state, and Nrf2 plays a crucial function in ROS-mediated TLR4 activation and in regulating TLR4-driven inflammatory reactions [27]. Furthermore, both TLR and Nrf2 signaling are triggered during inflammation, and TLRs trigger Nrf2 signaling in reaction to inflammation [28]. A recent study showed that EA protects rats against mitochondrial dysfunction by upregulating Nrf2/HO-1 and inhibiting the NF-κB signaling pathways [29]. Therefore, we evaluated the expression levels of Nrf2 and TLR4 and their associated proteins in order to elucidate the mechanism behind EA’s protective capability against SD-induced oxidative stress injury and inflammation. EA effectively activated the Nrf2/HO-1 pathway and mitigated the TLR4-induced inflammatory response in our study.

Based on the similar studies using a model of Glu-induced injury of hippocampal neurons [30], we further investigated whether EA plays a protective role in Glu-induced hippocampal neuron injury via the Nrf2 and TLR4 pathways. TLR4 signaling cascades cause the activation of NF-κB and the induction of proinflammatory cytokines [31]. Activation of the transcription factor Nrf2 induces several downstream neuroprotective genes related to antioxidant enzymes to protect hippocampal neuronal cells [32]. In addition, studies have shown that crosstalk between Nrf2 and NF-κB reliant signaling regulates inflammation [31, 33]. Our study showed that EA significantly reduced the mortality and ROS levels of injured cells, and these effects of EA were enhanced in TLR4 siRNA-transfected neurons. However, knockdown of Nrf2 dramatically restrained the protective impact of EA on Glu-induced toxicity. The current findings suggest that the TLR4 and Nrf2 signaling are involved in modulating inflammation and oxidative stress-related responses in SD.

**Figure 8:**
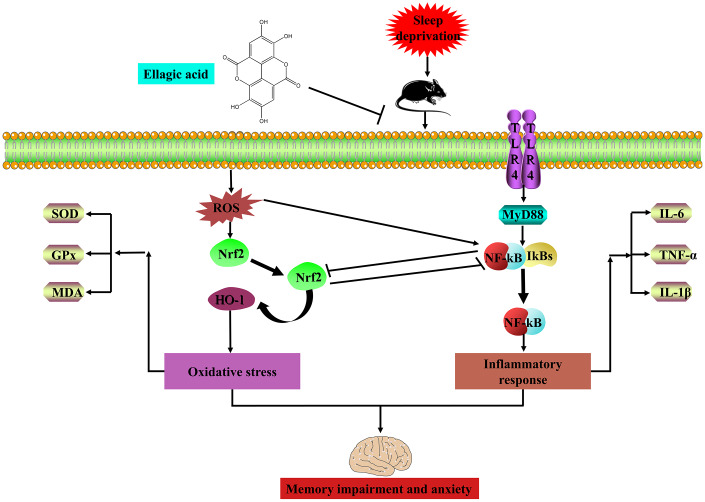
**EA ameliorates sleep deprivation-induced memory impairment and anxiety via crosstalk between the Nrf2 and TLR4 pathways.**

In conclusion, the present research indicated that EA protects mice against SD-induced cognitive impairment and anxiety by inhibiting TLR4 and activating Nrf2 (Figure 8). These findings suggested that EA is a prospective candidate for the prevention of SD-induced memory impairment and emotional disorders. Thus, EA may be a promising nutraceutical ingredient to prevent cognitive impairment and anxiety caused by sleep loss in the human population.

## MATERIALS AND METHODS

### Reagents and apparatus

EA (purity > 98%) was purchased from Xi’an Xiaocao Biological Technology Co. Ltd. (Xi’an, Shaanxi, China). IL-1β, IL-6, TNF-α, SOD, GPx and MDA commercial assay kits were from Nanjing Jiancheng Bioengineering Institute (Nanjing, China). The open field, elevated plus maze, novel object recognition, object location, Morris water maze and sleep deprivation apparatus were developed by Shanghai Yishu Technology Co. Ltd. (Shanghai, China).

### Animals and treatments

**Figure 9:**
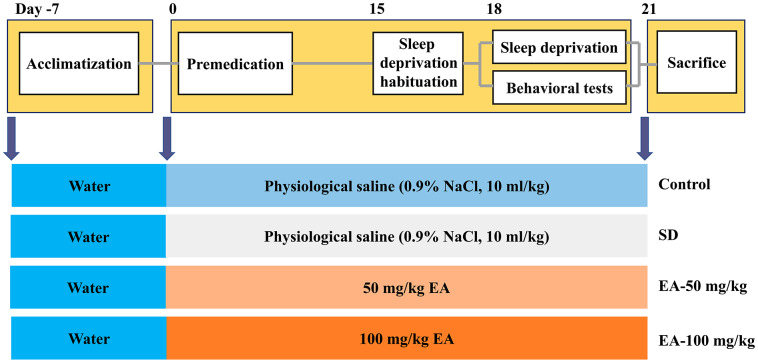
**Experimental design procedure. Mice were randomly divided into four groups after habituation for 7 days. Then, mice were administered EA daily intraperitoneally EA for 21 days.** After 3 days of SD habituation (from 8 a.m. to 11 a.m., 3 hours per day), all groups except the control group were subjected to SD for 72 hours (from 8 a.m. on day 18 to 8 a.m. on day 21). Behavioral tests were carried out after 24 hours of SD (Morris water maze training began on day 18).

The study protocol was approved by the Ethics Committee of Animal Experimentation of Fourth Military Medical University. C57BL/6J mice weighing 18-22 g were obtained from the Fourth Military Medical University’s animal care facility. All animals were kept in cages at room temperature (25 ± 1 °C) with free access to water and food. Mice were housed in a 12-hour light/dark cycle and left 7 days to acclimate before experimental procedures began. The mice were randomly assigned to four groups (n=12 per group): the control group, SD group, and SD treated with EA (50 and 100 mg/kg) groups. The mice were administered EA daily intraperitoneally for 21 days, and the control and SD groups received physiological saline (0.9% NaCl, 10 ml/kg, i.p.) at the same times. After 3 days of SD habituation (from 8 a.m. to 11 a.m., 3 hours per day), all groups except the control group were subjected to SD for 72 hours (from 8 a.m. on day 18 to 8 a.m. on day 21). Then, behavioral tests were carried out after 24 hours of SD (Morris water maze training began on day 18). Following the behavioral tests, the mice were sacrificed for biomarker assays (shown in Figure 9).

### Induction of the SD model

**Figure 10:**
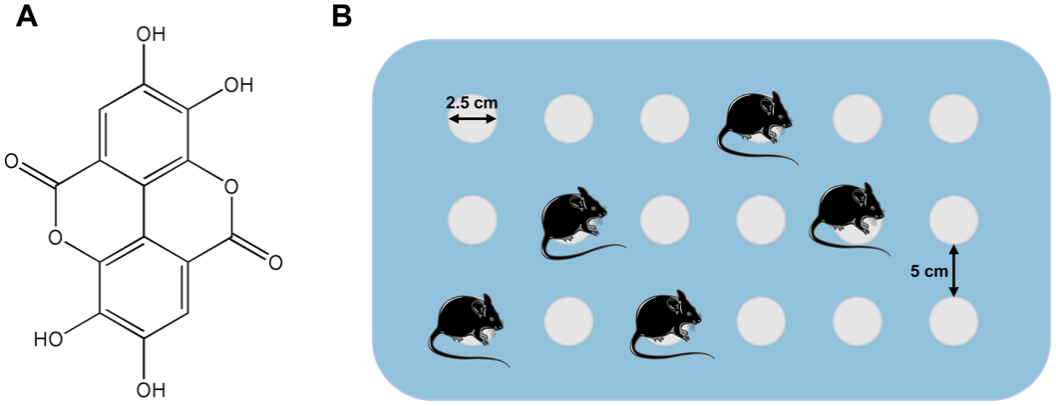
(**A**) Structure of ellagic acids (PubChem CID: 5281855). (**B**) Simple illustration of the modified multiple-platform method used for SD mice.

The model of SD was instituted using the modified multiple-platform method (Figure 10B), as previously described [34]. Eighteen columns (2.5 cm in diameter) were placed in a water tank (1 cm above the water level). The distance between the 2 columns was 5 cm so that the mouse in a water-filled bath could move freely on each platform by jumping. Thus, when the animal entered a sleep episode, it fell into the water and woke. The mice were continuously deprived of sleep for 72 hours, according to previous studies [35]. During the 72-hour SD period, the mice had free access to water and food. The control group mice were only kept in cages.

### Novel object recognition (NOR) and object location (OL) tests

The NOR was carried out to assess recognition and memory ability as described previously [36]. The experiment consisted of 3 phases (habituation, training and testing phase) (Figure 1A). Mice spent 10 minutes/phase for 3 days exploring the apparatus without any stimulus and each phase was recorded by a video tracking system. On day 1, the mice were placed in a chamber to adapt to the new environment (habituation phase). On day 2, the mice were permitted to explore the same 2 cylinders (training phase). On day 3, one cylinder was replaced by the same volume of cube and then the mice were allowed to explore (testing phase). In the object location (OL) test, the same device and software as for the NOR used in this test (Figure 1A). In the habituation phase, the mice were placed in an empty black plastic box to adapt to the new environment for 10 minutes. In the training phase, 2 cylinders were placed near the corner of the same wall. Each mouse was allowed to explore for 10 minutes. In the testing phase, one of cylinder was placed in the opposite position of the original position. Interaction parameters were specified as contact with the object (tail only excluded) or facing the object (distance < 2 cm). The “discrimination index” was calculated as the exploration novel object (location) time/total exploration time.

### Morris water maze (MWM) test

The spatial learning and memory of the mice were evaluated using the MWM with slight modifications [37]. A circular tub (60 cm in semidiameter and 50 cm in height) enriched by white opaque water (22-25°C) was divided into 4 equal-sized quadrants. Extramaze visual cues were placed in the 4 corners for spatial orientation. Mice experienced a training phase with the platform hidden in the target quadrant and a probe test phase without the platform. In the training phase, mice were trained for 4 days (60-second trial time, 4 trials each day with an approximately 20-minute intertrial interval) to seek the hidden escape platform (10 cm diameter, 2 cm below the water level). The entry quadrant varied but the platform location remained constant. The latency to find the platform was measured. If a mouse failed to find the platform within the maximally allowed time of 60-second, it was guided to the platform by the experimenter and allowed to remain for 15 seconds before being removed. A single 60-second probe trial was conducted with the platform removed after the final learning trial. The latency to the target area, time spent in the target quadrant, platform crossings and distance traveled were calculated. An automatic tracking system was used to record all behavior in real time.

### Open field test

The open field test was conducted to test locomotor activity [38]. Briefly, mice were placed in a square arena (30 cm × 30 cm × 30 cm) with clear Plexiglas walls and dim illumination. Then, all mice were allowed to freely explore for a 15-minute period. The mouse movements were recorded by a camera and analyzed with a video tracking system.

### Elevated plus maze (EPM) test

The elevated plus maze was conducted as described previously [39]. The apparatus comprised two open arms (25 cm × 8 cm × 0.5 cm) and two closed arms (25 cm × 8 cm × 12 cm) that extended from a common central platform (8 cm × 8 cm). Mice were allowed to habituate to the testing room for 2 days before the test and pretreated with gentle handling two times per day to minimize anxiety. For each test, an individual animal was placed in the center square, facing an open arm, and allowed to move freely for 5 minutes. The entrance was defined as all four paws placed inside an arm. The number of entrances and time spent in each arm were recorded.

### Hematoxylin and eosin (H & E) staining

After the behavior tests, the brains were fixed in cold 4% paraformaldehyde in 0.1 M phosphate-buffered saline. Coronal sections (20 μm) from the hippocampus were cut on a cryostat, and stained with H & E. The sections were observed to evaluate the morphological changes in the hippocampus under light microscopy (Olympus, Japan) after staining.

### Determination of pro-inflammatory cytokine and antioxidant enzyme activities in the hippocampus

For the biochemical assays, the mice were anesthetized and euthanized. Then, the hippocampus was quickly removed, homogenized and centrifuged (12000×*g*, 10 minutes, 4°C). The supernatants were collected for later experiments. Then, the levels of IL-6, TNF-α and IL-1β and the activities of SOD, GPx and MDA were measured by using commercial assay kits (Nanjing Jiancheng Bioengineering Institute, Nanjing, China). The final results were expressed as pg/mg tissue for IL-6, TNF-α and IL-1β and U/mg protein for SOD and GPx activities.

### Golgi-Cox staining and spine density analysis

As SD induced memory impairment and anxiety were confirmed, SD mice were subjected to Golgi staining as described previously [40]. Mice were anesthetized with pentobarbital sodium and brains were removed. Thereafter, brains were incubated in Golgi-Cox solution (1% potassium dichromate, 1% mercuric chloride, 0.75% potassium chromate) for 12 days at room temperature in the dark, followed by gradient ethanol dehydration. Coronal sections were sectioned (120 μm) using a vibratome. Hippocampal slices were collected on slides using neutral balsam and imaged on an Olympus BX51 light microscope using DP-BSW software with a 100x/NA 1.4 oil immersion lens.

### Western blot analysis

The hippocampus was homogenized in ice-cold RIPA lysis buffer. Then, the homogenate was centrifuged (12000×*g*, 10 min, 4°C), and the supernatant was collected. BCA protein assay kits (Pierce Biotechnology, Rockford, IL, USA) were used to determine the protein concentration. Protein amounts of 30 μg were electrophoresed and transferred to a polyvinylidene difluoride (PVDF) membrane (Millipore, Billerica, MA. USA). Then, 5% nonfat milk in 0.1% Tween 20 in TBS (TBST) was used to block nonspecific binding for 1 hour at room temperature. The blots were incubated overnight at 4°C with primary antibodies to anti-TLR4 (1:2000), anti-MyD88 (1:2000), anti-p-IκBα (1:2000), anti-NF-κB p65 (1:2000), anti-Nrf2 (1:1000), anti-HO-1 (1:1000) and anti-β-actin (1:10,000). Immunoreactive bands were detected by an enhanced chemiluminescence kit and imaged using a Tanon imaging system (Tanon 4200, China).

### Primary hippocampal neuronal cultures and treatment

Primary hippocampal neurons were prepared from embryonic d15 mouse embryos. Embryonic brain tissue was mechanically triturated and centrifuged. Neurons were cultured in an atmosphere of 5%/95% CO_2_/air at 37°C using the Dulbecco’s modified Eagle’s medium (DMEM) which contains 10% fetal bovine serum, 100 U/mL penicillin, and 100 μg/mL streptomycin. The coincubation model incorporating samples and Glu was used to evaluate the protective effects of EA on Glu-induced toxicity in cells. The equivalent volume of PBS was used in the control groups. All operations were repeated three times.

### Determination of ROS and cell viability

Cultured neuronal cells were transfected with TLR4 or Nrf2 siRNA (100 nM) (Santa Cruz Biotech) for 18 hours to examine the effects of gene knockdown in neurons. After recovery for 24 hours, the cells were treated with EA for 24 hours before being harvested for measurement of cell viability and intracellular ROS levels. Cell death was detected and quantified using the 3-[4,5-dimethylthiazol-2-yl]-2,5-diphenyltetrazolium bromide (MTT) assay.

### Statistical analysis

Data were analyzed using the GraphPad Prism 6.0 software package. All values are expressed as the mean ± standard error of the mean (SEM). Differences among all the groups were determined by one-way analysis of variance (ANOVA) followed by the least significant difference post hoc test. Significant differences were reported at *P* < 0.05.
